# Supplementary material for: Are carbon emissions trading and green financial instruments synergistic? -Comprehensive quantitative research based on content analysis
Source: PLoS One. 2024 Mar 7;19(3):e0298601. doi: 10.1371/journal.pone.0298601 (PMC10919601; doi:10.1371/journal.pone.0298601)
Supplement: S1 Data — (ZIP) [file pone.0298601.s001.zip › Data/地级市Z_F工作报告.zip!/║ú─╧/╚2╤╟╩╨/└┤╫╘╣1⁄2╓┌║┼í╛┬φ┐╦╩2╛▌═°í┐_2005.docx]

各位代表：

现在，我代表市政府向大会作政府工作报告，请予审议，并请市政协各位委员和其它列席人员提出意见。

2004年工作回顾

2004年，在省委、省政府和市委的领导下，在市人大、市政协的监督和支持下，市政府紧紧依靠全市人民，以科学发展观为指导，创造性地做好各方面的工作，圆满地完成了市四届人大二次会议确定的目标任务。

一、综合经济实力明显增强。去年是我市近十年来经济形势最好的一年。初步统计，全市生产总值48.25亿元（含农垦，下同），同比增长13.4%，其中一、二、三次产业分别增长7.8%、22.3%、14.5%；全市财政总收入8.87亿元，同比增长32.3%，其中地方财政收入5.38亿元，同比增长37%；全社会固定资产投资总额38.46亿元，同比增长41.9%；金融机构各项存款余额103.9亿元，比年初增长16.1%。

二、优势产业不断发展壮大。南田珠江温泉酒店、万豪度假酒店、天福源度假酒店、亚龙湾红峡谷高尔夫球场、肖旗港旅游码头等项目已建成营业，红树林度假酒店、金茂希尔顿大酒店、南山海上观音等项目进展顺利；以打击出租车、海鲜排档、桑拿按摩店欺客宰客为重点的旅游市场综合整治行动取得了阶段性成果；旅游市场促销成效显著，海外客源市场止跌回升，海外游客结构日趋多元化。我市已成为全世界最大的潜水旅游目的地、中国最重要的高尔夫旅游目的地和会议旅游目的地之一。去年全市星级饭店累计接待过夜游客364万人次，同比增长21.2%；旅游总收入39.14亿元，同比增长33.8%。旅游产业发展初步呈现出转型增效、协调发展的良好态势。我市克服了“尼伯特”台风和干旱对农业生产的消极影响，大力发展热带高效瓜果菜和花木产业，扩大优势水产品的养殖规模和农产品精深加工，突出抓好“十头万户”养猪、百艘百吨位渔船建造、兰花种植示范点等三大亮点工程，完成了宁远河南干渠、半岭果园节水灌溉项目和半岭、汤他等14宗水库除险加固工程，农业综合生产能力不断提高，农林牧渔业总产值达到30.27亿元，同比增长8.4%。加大了工业园区规划建设力度，初步建立起科技工业项目库，发电量、农产品加工、中成药、茶多酚等资源型产品的产量大幅度增长，工业经济运行质量进一步提高，全年工业总产值达到15.17亿元，同比增长7.9%。房地产业稳步发展，储备土地总量达到8065亩，全年“招拍挂”土地171.6亩，实现土地收益4.3亿元；完成商品房施工面积137万平方米、交易面积75万平方米、交易额31.1亿元，同比分别增长112.4%、50%、73%。

三、城镇建设再上新台阶。通过国际竞标和国际咨询的形式，完成了阳光海岸、鹿回头半岛、三亚湾新城、三亚凤凰国际水城等重点片区的规划设计工作；临春片区、月川新区、三亚学院等控制性详细规划编制和西岛建设规划修编也已完成。夏日百货等3个商场已建成营业；胜利路、鹿岭路等5条市区骨干道路改造工程和三亚河、临春河两座步行桥建设已完成；西岛、蜈岐洲岛、崖城水厂等3个供水项目已建成并投入使用；丰兴隆大桥、迎宾路改造、天然气管道、城市安全供水、污水管网二期扩建、凤凰机场二期扩建、国际客运港、河口旧城改造等新建和续建的基础设施项目建设进展顺利，村镇基础设施也不断完善。城市建成区新建绿地2.3万平方米，全市新增林地9817亩。全年共拆除违章建筑4.8万平方米，销毁非法营运车辆6000多部，取缔违章占道经营摊点近2000个，查处环境违法案件321起，“一控双达标”工作成效显著，城市环境综合整治取得良好效果。由于城市人居环境不断改善，我市去年被评为首届“最佳中国魅力城市”。

四、改革开放取得新进步。去年，我市被批准为全省第一个实行城市综合行政执法的试点城市；城市供水中外合作项目运行顺利，完成了北亚航空的国有资产重组，组建了国资控股的城市建设投资公司、国际旅游会展公司；推进政府非税收入征缴改革，把市级各类财政性收入纳入非税收入系统管理范畴；农村改革进展顺利，基本完成了农村土地确权工作，取消了农业特产税，下调农业税率一个百分点。农垦经济体制改革、金融管理体制改革、教育体制改革、社会保障和就业制度改革也取得一定进展。与美国毛伊县等7个国外城市签署了结好意向书，与乌克兰雅尔塔市缔结为友好城市。全年实际利用外资1.06亿美元；外贸进出口总额达到0.475亿美元，同比增长10.1%；交通邮电业快速增长，其中全社会货运量、客运量和邮电业务总量分别增长7.8%、10.3%和12.6%；凤凰国际机场全年进出港游客252万人次，同比增长48.8%。成功举办了首届中国青年欢乐节暨第五届海南岛欢乐节闭幕式、第54届世界小姐总决赛等重大节庆活动，三亚的美誉度、知名度和吸引力均得到空前提升。

五、人民生活水平稳步提高。文明生态村建设和农村扶贫成效显著，修建了乡村“11路1桥”，建设文明生态村40个，扶贫种植橡胶9万株和养猪5000头，新建沼气池2560个，完成民房改造1158户，改厕改水1323户，解决了农村2万人饮水难问题。城市“低保”覆盖面不断扩大，“两个确保”率巩固在100%，全市9921名离退休人员均按时足额发放养老金，为全市低保对象7451人发放了基本生活保障金1152万元，为3827名农村特困对象发放了救助金159万元，为164名困难企业军转干部解决了生活保障问题，为6500多名农民工追回被拖欠的工资809万元。全市新增城镇就业岗位12664个，同比增长41.8%，城镇登记失业率为2.8%，就业工作取得新的突破。全市在岗职工平均工资13000元，同比增长12.4%；农民人均纯收入达到3157元，同比增长7%；城乡居民储蓄存款余额55.92亿元，比年初增长17.3%；全社会消费品零售总额18.12亿元，同比增长22.4%，城乡居民消费水平不断提高。

六、社会事业全面发展。2004年是我市历史上对科教文卫事业投入最多的一年，也是发展最快的一年。“国家农业863海南研发基地”项目已基本建成，“中国三亚农业科学城”项目正在积极推进之中。旅游航空学院、海南鲁迅中学已建成并投入使用，三亚市一中新校、市第九小学等项目进展顺利；对全市19个省级贫困村7117名小学生实行免费入学，全市小学适龄儿童入学率达到99.9%，初中生入学率达到98.2%。体育中心、市民网球馆等项目已动工，三亚电视台第一套新闻综合节目已覆盖了全省19个市县，全市农村广播电视覆盖率已达到95%以上，全民健身运动等群众性文体活动蓬勃开展。天涯、高峰等8个卫生院改造项目已完成，市人民医院改扩建工程、市中医院新区等项目进展顺利，初步建立起覆盖城乡的医疗急救体系网络，全年为贫困群众义诊及健康咨询28580人次。全市人口出生率12.9‰，自然增长率为9.5‰。社会治安综合治理成效显著，市场经济秩序继续好转，安全生产进一步加强，社会政治保持稳定。国防动员、民兵预备役工作及拥军优属、拥政爱民活动深入开展，军政军民团结进一步加强。统计、科协、民族宗教、防震减灾、人防、工会、共青团、新闻出版、档案、修志及红十字会、妇女儿童、老龄、残疾人等方面工作都取得新进步。

七、政府自身建设得到加强。开展了“三优四满意”活动，提高了政府服务水平。加大了廉政建设力度，全年查办违法违纪案件50宗，挽回经济损失223万元；完成审计项目38个，查出违法违规及管理不规范资金1.61亿元,经审计处理后上缴财政6056万元，核减市政工程造价2380万元。全年出台了24个规范性文件，修改和废止55件规范性文件，取消了65项行政审批事项，提高了政府依法行政能力。成立了市机关效能投诉中心，建立起行政过错追究制度和投诉处理机制。扎实推进民主政治建设，严格执行市人大及其常委会的决议、决定，定期向市政协常委会通报政务情况，全年办复了市人大代表建议和议案59件及市政协委员提案97件。加强同各民主党派、人民团体的联系，虚心听取各界人士的建议、意见。认真为民办实事好事，去年确定的11大项共68件实事好事已基本上兑现。

各位代表，过去的一年，我们千方百计抓机遇，一心一意谋发展，实现了国民经济持续健康快速发展和社会全面进步，取得的成绩令人振奋，这是全市人民共同努力和各方面大力支持的结果。在此，我谨代表市政府，向全市人民、人大代表和政协委员，向各民主党派、人民团体和各界人士，向离退休老同志，向中央和省驻三亚单位，向驻三亚部队和武警官兵，向所有来三亚参加建设的投资者和劳动者，向一直关心支持三亚发展的海内外朋友，表示崇高的敬意和衷心的感谢！

回顾去年的工作，我们也清醒地看到，在前进中我们仍面临着许多困难和问题，政府工作还有不少缺点，人民群众还有一些不满意的地方，主要表现在：一是经济基础薄弱，经济总量小，城镇化水平低，城乡居民收入水平仍然偏低。二是经济社会发展不够协调，社会事业发展滞后。三是城市规划、建设、管理和经营的理念与建设“一市两都三中心”的目标还存在较大的差距，城市规划在执行中有偏差,城市管理仍存在盲区。四是农业抵御灾害能力不强，农民收入来源缺乏稳定性，增收缓慢。五是海滩泥化、虾塘污染和毁林开荒等破坏环境资源的现象未能得到有效解决，经济发展与环境保护的矛盾依然较为突出。六是就业形势依然严峻，社会保障能力较弱，部分群众生活困难。七是因征地拆迁、土地纠纷、企业改制、交通混乱、拖欠农民工工资等引发的社会矛盾和不安定因素有所增多。八是政府作风建设形势不容乐观，投资环境仍不尽人意，行政不作为、行政乱作为和“吃拿卡要”等现象，在个别单位和少数公职人员身上依然存在。我们将高度关注这些问题，并采取有效措施在今后的工作中努力加以解决。

2005年工作安排

2005年是“十五”计划的全面实现年，也是“十一五”规划的基础年，我们要全面完成“十五”计划，科学谋划“十一五”规划。根据省委、省政府的总体要求和市委的部署，今年政府工作的基本思路是：以邓小平理论和“三个代表”重要思想为指导，认真贯彻落实市委四届三次全会精神，坚持以人为本，以科学发展观统领经济社会发展全局，坚定不移地实施“一市两都三中心”战略，着力提升城市核心竞争力，大力推进经济结构调整，培育和壮大优势产业，加快体制改革和扩大开放，促进经济增长方式转变，坚持把维护和发展人民群众的根本利益作为政府工作的出发点和落脚点，推动社会事业全面进步，努力构建“和谐三亚”，实现经济社会跨越式发展。

根据上述要求，综合考虑各方面因素，建议今年我市国民经济和社会发展的主要预期目标为：全市生产总值增长14%以上；地方财政收入增长18%以上；全社会固定资产投资增长17%以上；社会消费品零售总额增长15%以上；实际利用外资增长11%以上；城镇居民年人均可支配收入增长9%以上，农民年人均纯收入增长7%以上；城镇新增就业岗位10000个以上，城镇登记失业率控制在4%以内；人口自然增长率控制在10‰以内。以上预期目标是积极稳妥、留有余地的，在实际工作中我们将根据执行情况适时提出新的要求，力争把各项工作任务完成得更好。

经过十几年的开发建设，三亚已成功地完成了从一个滨海小镇向专业化旅游城市和中国首选的休闲度假旅游目的地的角色转变，目前正处于快速发展期和经济发展上升期，已具备加快发展的条件和潜力。市委与时俱进，确定了符合三亚实际的“一市两都三中心”的城市发展战略目标，三亚的发展思路比过去任何时期都更加清晰和稳定。当前，国际国内经济形势总体趋好，我国也已进入以工促农、以城带乡的发展阶段，我市经济发展仍是有利条件多于不利条件，只要我们审时度势，趋利避害，努力发挥优势，就一定能保持我市经济社会持续快速发展。今年的政府工作，将重点抓好以下六个方面：

一、加快城镇化进程，进一步提升城市品位。对三亚而言，城镇化是三亚加速发展的动因，是建设国际性热带滨海旅游城市，加快农村人口转移，实现小康目标的重要举措。

进一步完善城市配套设施。重点加强城市基础设施建设，今年固定资产投资优先放在公共基础设施和基础产业等重点推进项目上。一是抓好凤凰机场二期扩建、白排客运港、225国道天涯至南山段改造、迎宾大道改造、一环路延伸段改造、丰兴隆大桥、红沙大桥等项目建设，做好南山货运港、环岛高速公路三亚连接段、鹿回头海景大道等项目的前期工作，进一步完善城市交通基础设施。二是加快城市污水管网改扩建、城乡电网改造、城镇供水、燃气管道等工程建设，进一步提高市政设施的覆盖率。三是完成迎宾路、凤凰路等景观绿化改造工程，加快两河三岸和三亚湾沿岸的靓丽工程建设，进一步扩大城区绿化覆盖率。

推进城乡一体化建设。围绕着“一片一线五点”的城市发展思路，统筹兼顾地开发建设专业化旅游城市。一是不断完善城市总体规划和各项概念规划编制及规划控制，尽快完成旧城区改造规划，加强海坡开发区、亚龙湾旅游区的规划控制，重新修编天涯海角景区规划，尽快完成落笔洞、红塘湾、海棠湾旅游区和南山开发区的总规编制。二是重点将海坡、鹿回头、临春山水国际度假村、三亚湾新城、时代海岸、阳光海岸及月川新城区，打造成为集生活居住、文化娱乐、休闲度假为一体的现代综合型社区。三是按照省委、省政府提出的城乡互动、三次产业联动的要求，加快五镇总体规划的实施，集中力量推进小城镇建设。要把小城镇建设同壮大区域经济、文明生态村建设、移民搬迁和改善群众生活条件结合起来。重点完成大隆水利枢纽工程移民安置、田独镇政府搬迁和居民新区项目的建设，加快田独龙坡沟家庭旅馆村、凤凰镇黎家文明生态村、亚龙湾湖滨商业街等小城镇产业群建设，构建优势互补的城镇布局体系，进一步推进农村城镇化。今年我市城市规模要扩大2平方公里以上，非农业人口要增加2万人以上。

构建城市管理长效机制。目前，我市的城市管理水平和城市定位与城市知名度极不相称，城市管理问题已成为制约三亚发展的突出问题。今年是我市的“城市管理年”，要创新城市管理体制，加大城市环境综合治理力度，向城市管理要效益，不断提升城市品位。一是出台“三亚城市规划管理标准和技术标准”，明确三亚的城市建筑风格及基本色调，做到不符合控制性详规的建设项目一律不予审批，重大的城市建设项目要在立项阶段广泛听取专家学者和社会各界的意见。二是从公共基础设施、防震减灾、园林绿化、环境卫生、交通管理、市民行为等方面入手，制定符合三亚实情的城市管理标准化体系，对城市逐步推行规范化和法制化管理。三是改革城市管理体制，重点推进城市综合行政执法试点工作，成立城市管理综合行政执法局；加快推进市政公用事业单位体制改革，园林局和环卫处下属单位改制为公司的工作要在年内完成。四是以违章建筑整治、交通秩序整治、旅游市场综合整治、资源环境整治和社会治安综合整治为重点，对城市环境进行综合治理，全面提升城市管理水平。在整治行动中，除了要对违法违章的单位及个人进行重处重罚和曝光外，还要从严查处公职人员非法参与或在其中充当保护伞的违法乱纪行为。

二、大力发展优势产业，不断壮大经济总量。要充分发挥市场机制的配置作用，不断转变经济增长方式，加快经济结构调整和优势产业升级。

深化旅游产业转型增效。今年要完成《三亚市旅游发展总体规划》编制工作和出台《三亚旅游国际化启动方案》，大力创新旅游行业的经营管理机制，不断拓展游客消费的领域和空间，力争全市星级饭店接待过夜游客达到425万人次和旅游总收入达到46亿元，同比分别增长16.5%和17.5%。为此，一是要着力打造名牌景区景点。加快天涯海角、亚龙湾、南山、西岛、大小洞天等重点景区景点的开发建设，南山海上观音、爱心大世界今年要建成开园，天涯海角游览区扩建工程、南山文化二期工程、海洋世界等项目要争取早日动工。二是精心打造品牌度假酒店。金茂希尔顿、红树林等7家度假酒店要在年内建成营业，凯悦度假酒店、天域度假别墅等3家度假酒店要在今年动工，我市五星级酒店在年内要达到15家。三是加大旅游产品开发力度。开辟“海上游三亚”的旅游线路，进一步开发和包装涉海娱乐、高尔夫休闲、民俗风情、温泉疗养等竞争力强的特色旅游产品，推出适合游客夜间消费需求的酒吧、演艺吧、休闲茶吧等娱乐产品，扶持开发椰雕、贝壳、珍珠、水晶、黎族纺织品、旅游景观纪念品等具有地方特色的旅游商品。四是加大旅游市场促销力度。政府将投入1500万元促销经费，整合旅游企业的资源和产品，把城市形象宣传和旅游产品营销有机结合起来促销，突出三亚旅游的整体品牌；充分利用互联网、有线电视网、移动通讯网，构建起自助旅游服务体系，为游客提供快捷的旅游信息咨询服务。五是加大旅游行业管理力度。大力整治旅游市场秩序，强化旅游市场综合监管执法，重点开展旅行社、导游和旅游购物、餐饮、娱乐行业的清理整顿，严厉查处对外承包经营、非法变相转让经营许可证、哄抬标价、敲诈游客等违法经营行为，建立健全咨询投诉、安全急救、散客服务等旅游保障服务体系，确保游客消费安全。六是针对境外游客大量涌入和自驾车游、散客自游人异军突起的发展趋势，由政府投资在年内建成国内一流的游客到访中心和旅游公共危机处理中心。七是加快旅游饭店的星级评选和景区景点的等级评定，继续引进国际知名酒店管理集团参与酒店业管理，加强旅游从业人员的技能培训，提高旅游行业的管理服务水平。

加快发展现代农业。要用工业文明的思想和理念改造农业，加快推进农业产业化、标准化和现代化进程，确保农村发展、农业增效、农民增收，力争全年农业增加值同比增长12.5%以上。为此，一是要大力发展热带高效农业和花木产业。对传统瓜果菜产品进行改良，扩大种植竞争优势强、效益高的冬季瓜菜和热带水果及花卉，全年瓜菜种植面积要完成19万亩，热带水果种植总面积达到19.6万亩，兰花种植总面积达到1000亩。二是大力发展畜牧业。重点发展养猪业和养鸡业，力争全年畜牧业产值同比增长16%以上。三是大力发展海洋渔业。全面完成百艘百吨渔船最后34艘的建造任务并下海作业，加快六道湾渔港等渔业基础设施建设，组建远洋捕捞船队到西沙、南沙开发新渔场，继续调整水产养殖结构布局，扩大优势水产品养殖规模，力争全年渔业产值同比增长13.5%以上。四是大力推进农业产业化进程。今年科技设施农业推广面积要达到6.5万亩；重点引进和建设一批上规模、上档次的畜牧业和渔业龙头企业，扩大“公司+基地+农户”的生产规模；着力推进农业基地化生产，扶持和培育一批高科技农业示范基地、农科教结合基地，加快“国家农业863海南研发基地”、“中国三亚农业科学城”、万亩超级水稻基地、海棠湾哈密瓜基地等项目和各类农产品综合加工厂建设。五是抓紧建立农业标准化体系。尽快制定和完善主要农产品的质量标准体系、检验检测体系和产品评价体系，扩大无公害农产品、绿色食品的认证范围，扶持建立一批农业标准化生产综合示范区和实施标准化管理的试点企业。六是搞活农副产品流通。引资建设三亚农副产品综合批发市场，政府出资在北京等城市建立农产品营销网络，扩大三亚农业信息网络平台的覆盖率。

积极发展新兴优势产业。要围绕着旅游产业大力发展新兴产业：一是加快发展新型科技工业。经济园区化、园区产业化是沿海地区经济发展的重要经验。要加快推进荔枝沟技工贸易区、田独高新技术产业园、三亚创意产业园的建设，对新型科技工业实行产业倾斜政策，大力发展资源加工型、生态环保型、高新技术型的新型科技工业项目，优先推进农副产品加工、生物制药、电子信息、旅游商品制造等发展项目，拉长资源型工业的产业链。政府将拨出500万元设立高科技产业风险基金，并创办国资控股的科技工业投资公司，力争全年工业增加值同比增长17.5%以上。二是稳健发展房地产业。进一步储备新的建设用地，适度控制非农用地的供给，力争土地储备总量达到1.2万亩；做好国有土地的“招拍挂”工作，全年完成土地收益4亿元。要对房地产业进行整体规划，提高房地产开发项目的审批门槛，继续实行适度发展、成片开发的政策，杜绝开发小而散的楼盘，着重发展休闲度假置业型的旅游房地产，同时兼顾市民改善住房条件需要，努力提高居民的住房质量，促进我市的房地产业由粗放型增长向集约型增长转变。全年要完成房屋新增建设面积100万平方米、交易面积100万平方米的“双百万”目标任务。三是做大做强文化健康产业。今年除办好传统旅游节庆活动外，重点做好世界级的文化盛典??――南山海上观音开光典礼活动、中国国际电视广告金椰子奖活动、第十四届中国电影金鸡百花奖暨中国电影诞辰100周年纪念活动，以及第55届世界小姐总决赛。要争取国际电影节、热带兰花国际博览会、城市街道汽车赛、国际摩托艇大赛、TCL高尔夫球精英赛等国际赛事和活动落户三亚，通过旅游节庆活动带动现代服务业、会展业和文化健康产业的发展。加快引进上海家化三亚温泉健康城、首都机场公司三亚半岭温泉疗养度假区等康复疗养项目，使文化健康产业成为新的经济增长点。

三、加快体制机制创新，增加经济发展活力。我市体制改革将全面进入攻坚阶段，要继续加大力度，做好改革的总体指导和统筹协调等工作，扎实有效地推进重点改革和对外开放工作。

招商引资要有新突破。一是转变招商引资的理念，将“招商引资”变为“招商选资”，对于带动发展能力小的投资项目，坚决拒之门外；对于促进就业小的投资项目，严格控制土地、税收、金融等优惠政策。二是提高投资项目的准入门槛，对一些事关我市长远发展的行业实行准入制，围绕我市基础设施建设和优势产业发展，做好招商项目的筛选工作，继续增加招商项目库的储备量。三是组织好农林水利、生态环境、基础设施等建设项目的上报工作，积极争取国债资金支持。四是在政府和企业两个层面上创新招商融资机制，瞄准市场投资需求，重点推出一批符合三亚发展需要且市场竞争力强的招商项目，吸引一批大项目、大企业在三亚落户，争取几个集团公司在三亚设立区域性总部。

对外开放要有新局面。一是制定友好城市和国外引智工作规划，今年要选择2个有利于推动我市经济社会发展的外国城市缔结为友好城市，引进40?―50名外国经济类专家。二是与境外媒体机构建立良好的合作关系，组织国际知名媒体组团来访，积极在国际上宣传、推介三亚休闲度假旅游形象。三是利用海南开放第三、四、五航权的机遇，增开2条以上国际航线和引进1个以上国际著名旅行社，大力拓展国际旅游市场，力争凤凰机场今年进出港游客达到350万人次。四是借助“双大互动”战略、“9+2”区域合作机制和各类涉外商贸活动，实施“走出去”战略，参与区域经济圈的互动合作，不断扩大对外开放。

非公有制经济要有新发展。一是认真贯彻落实《海南经济特区促进私营个体经济发展条例》，鼓励社会民间资金和外资依法参与我市经济建设，放宽非公有资本进入企业重组改造、城市基础设施建设、公用事业、社会事业以及国家没有明令禁止的产业和领域。二是引导个体私营经济朝外向型、科技型和多元化、规模化的方向发展，提高非公有制经济的比重和水平。鼓励个体私营经济组建企业集团，扶持一批骨干企业，形成规模经济参与市场竞争。三是继续抓好整顿和规范市场经济秩序工作，加大食品、药品、医疗、生猪屠宰、农资、成品油、汽车交易、废旧物资、烟草专卖、文化娱乐等市场的监管执法力度，为非公有制经济创造良好的市场环境。

财税金融改革要有新成绩。一是加强税收征管，积极拓展财源。重点加强旅游行业、房地产、房屋出租、汽车市场、医药购销企业、土地增值税的税收管理；依法清理和规范税收优惠政策，严格控制减税范围，做到应收尽收，确保今年完成地方财政收入三年翻一番的目标任务。二是加强财政预算管理，提高项目预算编制的科学性和效益性。继续深化国库集中支付管理制度改革和政府非税收入征缴改革，扩大市本级预算单位实行财务集中核算试点工作，并将政府非税收入征缴系统推广到镇级；加强对市本级财政预算执行审计监督和财政效益评价。三是全面厉行节约，合理安排财政支出。要严格控制一般性支出增长，逐步减少经营性事业单位的财政拨款，增加社会保障支出，对农业、就业、社保、公共卫生、科技教育、生态环境建设等领域加大财政投入，把有限的财力用在刀刃上，提高财政资金使用效率。四是加强财政监督管理，努力防范和化解政府债务风险。五是支持金融机构深化改革和加强监管，加大金融对地方经济发展的支持力度。

国资国企改革要有新成效。一是不断完善国资监管新体制，探索和建立国有企业预算管理制度、企业经营业绩考核制度和产权代表委派制度，建立起国有资产经营责任制。二是大力推进现代企业制度建设，加强产权代表工作，逐步完善市属重点国有企业及国有控股、参股企业的法人治理结构。三是整合我市国有经济的布局和结构，扶持天涯水业集团公司、市国资公司、市旅游投资公司、三亚港集团公司、国际旅游会展公司、城市建设投资公司、西岛旅游开发公司等企业不断发展壮大，做强做大国有经济。四是加快国有企业经营机制转换，规范国有企业改革工作流程和企业国有产权转让行为，严防国有资产在重组改造的过程中流失；国有企业进行重组改造，要以做好职工安置为前提。五是开展市属经营性事业单位的国有资产清产核资工作，加强国有资产的收益收缴工作，力争国有资产年收益达到4800万元以上。

农村和农垦改革要有新进展。建立符合农民利益的土地流转机制，健全农村土地征用赔偿安置机制，推行“土地换社保”政策，切实解决失地农民安置问题。按省的要求从今年起全部取消农业税。积极推进地方和垦区经济社会资源的整合，做好农垦系统困难职工城市低保工作，把农垦离退休人员养老保险统一纳入市级统筹范围，利用农垦先进的农业技术为地方服务，探索地方和垦区的教育、医疗等社会职能如何实现属地化管理，充分发挥农垦对三亚经济社会发展的重大推动作用。

人事制度改革要有新举措。一是要尽快出台事业单位人事制度改革的配套文件，指导改革工作稳步推进。二是推行事业单位人员聘用合同制，引入激励竞争机制，形成人员能进能出、职务能上能下、待遇能升能降的科学用人机制。三是根据教育、科研、文体、卫生等各类事业单位的特点，制定符合管理、专业技术和工勤岗位要求的管理制度，建立科学分类的管理体制。四是抓紧制定人才工作规划，坚持培养人才和引进人才并重，大力引进高层次管理人才和紧缺人才，建立健全人才激励机制和效益分配制度。五是取消事业单位的行政级别，不再按行政级别确定事业单位人员的待遇。今年要完成教育、卫生等4家事业单位的人事制度改革试点工作和超编事业单位人员的分流工作。

四、切实做到统筹兼顾，增强可持续发展能力。要按照科学发展观的要求，统筹推进经济与社会、城市与农村、人与自然的协调发展。

大力发展社会各项事业。我市社会事业发展滞后，已成为制约我市经济发展的瓶颈。为统筹经济社会协调发展，今年我市将继续加大教育投入，实行教育投资多元化，三亚学院、三亚市一中新校、第九小学要在年内建成并招生，力争今年中小学新增8000个学位，高校招生人数达到3000―5000人；调整全市中小学校布局，扩大优质初中、高中规模；整合农村教育资源，扶持办好市民族中学，争取在3年内完成全市中小学校的危房改造任务。加快科技基础设施和电子政务、电子商务的建设，提高科技创新能力，今年要以全市党政电子政务网络系统建设为重点，健全科技推广网络，推进我市信息化进程。加强崖城孔庙、落笔洞等文物资源的保护和开发利用；大力推进市体育中心、影视主题公园、国际会展中心等骨干项目建设；举办好亚洲职业高尔夫三亚公开赛、国际铁人三项赛等体育比赛活动，广泛开展全民健身、广场文化、文化下乡等群众性文体活动，提高全民健康水平。加快医疗卫生基础设施建设，市人民医院改扩建一期工程、中医院新区项目、市防疫站改造工程要在今年建成并投入使用；不断加强疾病预防控制体系、医疗急救体系建设，加快农村卫生事业发展。广泛开展国防教育，加强国防动员和民兵预备役工作，注重提高双拥共建质量，努力争创国家双拥模范城。进一步做好防震减灾、红十字会、妇女儿童等工作，大力发展老龄事业，关心残疾人的工作和生活。

加大对“三农”工作的投入。我市城乡二元经济结构明显，城乡差距较大。为统筹城乡发展，一是加快农村人畜饮水工程建设。兴建槟榔村、盐灶村等3宗人畜饮水工程，解决农村1.3万人饮水安全问题。二是大力建设农田水利设施。重点推进大隆水利枢纽工程建设，对水源池等水库进行除险加固，对汤他、抱孟、宁远河、青田等灌区进行水渠硬化，大力整治力理田洋、崖城白超――大坡田洋、海棠湾落根――文针田洋，增加灌溉面积2.44万亩，改善和恢复灌溉面积3.48万亩。三是大力开展农民科技培训和服务活动。全年免费对农民进行农业实用技术培训要达到2万人次；在全市建立“农村科技服务110”网络系统，争取在各区镇、农场都建立6―8个“农村科技服务110”服务站。四是实施农村劳动力转移工程。着手改革城乡户口分治的户籍管理制度，放宽农民进城落户条件，鼓励农民进城务工和定居，加快推进农村富余劳动力向城镇转移。今年要成立农村劳动力转移综合服务机构，并组织3000名农民进城务工。五是继续实行良种补贴、渔民造大船补贴和农机购置补贴政策。

做好人口、资源和环保工作。三亚生态环境中国一流，这是我市最大的本钱和优势所在。为统筹人与自然协调发展，今年我市要全面落实人口与计划生育目标管理责任制，加强农村人口和城市流动人口的管理，抓好孕育节育措施的落实，促进优生优育。依法加强资源管理，积极推进生态节水型城市建设，继续加强“三湾”整治，全面查处非法使用海域的行为，彻底整治三亚湾沙滩泥化和海棠湾海滩的虾塘养殖污染，从重打击非法抽取地下水、乱挖河沙、开山采石、烧山毁林、破坏珊瑚礁、捕杀珍稀野生动物等违法行为。加大生态环境建设和保护力度，在更高层面上推进生态市建设，重点抓好退塘还林、营造“三边林”和山体绿化恢复工程，恢复种植海岸带的红树林，严厉打击各种乱排污水行为，全面查处城市噪声污染和油烟污染。加强开发建设项目的环境监管，遏制新污染源的产生。

五、坚持以人为本，构建“和谐三亚”。我们目前正处在经济结构调整、体制转型和社会变革的关键时期，要解决好关系群众切身利益的问题，稳定经济社会发展的大局。

积极为民办实事好事。一是大力做好就业再就业工作。统筹指导大中专毕业生、复转军人和城市新增劳动力进行就业，继续利用再就业优惠政策扩大就业容量，确保今年新增就业岗位10000个，下岗失业人员通过劳动力市场实现再就业350人，对外输出劳务1000人。二是进一步完善社会保障体系。扩大城乡五项社会保险的覆盖面，巩固“两个确保”，继续推动下岗职工向失业保险并轨，实现养老、医疗、工伤、生育和失业保险管理“五险合一”；将地方政府接收安置的部队退休职工纳入医疗保险范围。三是建立城乡低收入困难群众社会救助体系。今年将设立贫困人口医疗救助基金和困难群众子女上学资助基金，政府出钱帮助困难群众解决看病就医、子女上学问题，全面启动新型农村合作医疗试点工作，从今年起全部免除农村中小学生义务教育的学杂费，继续扶助19个省级贫困村学生进行免费教育，并扩大边远贫困地区的免费教育面。四是加大扶贫开发力度。建设文明生态村40个、沼气池2000户，完成民房改造1000户、改厕改水2100户，修建乡村公路11条，种植扶贫经济作物6000亩，扶持3000户贫困农民养猪1万头。五是严格执行最低工资制度，着力解决企业拖欠职工工资问题；严厉查处拖欠工程款和农民工工资的行为，政府以往所拖欠的工程款要尽快解决，本届政府保证不出现新的拖欠行为。六是尽快建立符合我市实际的农村干部退休保障制度，解决农村干部的后顾之忧。

加强精神文明建设。坚持社会主义物质文明和精神文明一起抓，实施公民道德建设工程，不断加强社会公德、职业道德、家庭美德教育，重点抓好未成年人的思想道德建设。加强科普工作，倡导科学、健康、文明的生活方式，提高市民文明水平。广泛开展文明安全社区、文明规范行业和文明诚信企业创建活动，大力推行城乡居民文明规范，进一步提高城乡文明程度。大力推进“诚信三亚”建设，不断提高三亚的公信力。

加强政治文明建设。各级政府要遵守国家法律法规，严格执行市人大及其常委会的决议、决定，自觉接受人大的监督。坚决支持市政协履行职能，加强与各民主党派和各界人士的联系，充分发挥工会、共青团、妇联等人民团体的桥梁和纽带作用；建立健全民主决策制度、专家咨询制度、政府重大决策公示制度和社会公共事业听证制度，保障公民对社会事务的知情权、参与权和监督权。大力推进社区建设，加强基层民主管理，完善村民自治制度，保证人民群众依法行使民主权利。健全法律援助体系，保护弱势群体合法权益。抓好普法教育，着力建设“法制三亚”，不断强化公民法制观念。

依法管理民族宗教事务。全面贯彻党的民族政策，巩固和发展平等、团结、互助的社会主义民族关系，处理好不同民族之间的矛盾，搞好民族团结。加大对民族地区经济社会发展的支持力度，加强少数民族各类人才培养，促进各民族共同繁荣发展。全面贯彻党的宗教信仰自由政策，依法管理宗教事务，积极引导宗教与社会主义社会相适应。坚决取缔非法宗教活动和基督教私设的聚会点，防止境外敌对势力和非法宗教势力的渗透。

切实做好社会稳定工作。建立健全社会预警体系和应急机制，落实社会治安综合治理责任制和责任追究制。严厉打击黑恶势力、严重暴力犯罪、“两抢一盗”、毒赌黄、“法轮功”等危害社会安全的行为，加强流动人口和易发生违法犯罪的行业及场所的管理，减少各类案件的发生，确保人民群众生命财产安全。各级政府、各部门都要高度重视人民群众来信来访工作，以“发现得早、化解得了、控制得住、处置得好”为目标，既要维护人民群众的合法权益，又要坚持依法办事，按政策办事。按照属地管理、分级负责和谁主管、谁负责的原则，建立健全各项工作机制，提高基层组织的执政意识和执政能力建设，力争把矛盾化解在萌芽状态，把问题解决在基层，预防群体性上访事件发生。加强物价管理，把物价控制在预期目标之内。完善安全生产管理机制，预防和减少重大事故。

六、加强政府自身建设，提高行政管理效能。行政不作为和乱作为行为以及行政效能低下等“机关病”，已成为我市经济发展的障碍。今年是我省“投资发展环境建设年”，要全面推进依法行政，不断提高政府行政能力，解决好政府自身存在的问题。

加强政府行政能力建设。一是大力转变政府职能。加快推进行政体制改革，加强作风建设，建立起有限型政府、法制型政府和服务型政府，进一步理顺政府与企业、政府与市场、政府与社会的关系，把政府经济管理工作着力点放在为市场主体提供优质服务和创造一流投资发展环境上来，吸引投资者主动前来投资。二是改变政府管理方式。政府要改变“以批代管”、“只批不管”的做法，尽快从干涉微观经济活动转移到经济调控、市场监管、社会管理的公共服务上来，将不该由政府管、管不了、也管不好的事，交给企业、市场和中介机构。三是继续清理不合法的行政许可事项，减少审批范围，规范办事程序，增加工作透明度，提高办事效率；坚决落实鼓励投资的优惠政策，切实降低企业投资成本；确定各部门主要领导为投资环境的第一责任人，进一步健全重点推进项目领导跟踪负责制，解决好损害投资者合法权益的问题。四是进一步理顺市、镇、村各级管理关系。把能够下放给区镇管理的事项下放给区镇，方便群众办事。

强化行政效能监察。一是加大行政监察和审计监督力度。完善预防腐败的行政监察和审计监督机制，对实施行政行为的全过程进行有效制约监督，着力规范行政审批、执法检查、收费征税、行政强制、立案处罚等行政权力的运作规程，防止有令不行、刁难拖延、推诿拖拉和浪费行政资源等问题发生。监察部门对重大活动和重点项目要进行行政监察，市机关效能投诉中心要深入一线加强日常性监督检查。二是大力惩治腐败行为。严肃查处以权谋私、权钱交易、“吃拿卡要”、与民争利，以及乱收费、乱摊派、乱罚款等违法乱纪行为；对于弄权勒索、徇私枉法等败坏政府形象的害群之马，要坚决绳之以法，绝不姑息！三是充分发挥社会舆论监督作用。欢迎社会各界对政府工作进行监督，对于任何违法违纪的人和事，新闻媒体要坚决进行曝光，三亚不允许有特殊公职人员存在。四是建立科学的政绩评价体系。坚决制止劳民伤财的形象工程、政绩工程和盲目建设行为，进一步完善决策目标、执行责任、考核监督体系，强化对部门、单位主要领导的经济责任审计和综合绩效考评。五是切实落实工作过错责任追究制和行政首长问责制。对于欺上瞒下、弄虚作假、浮夸虚报和工作不得力、群众反映强烈的单位，以及让三亚错失发展机遇的人，从严追究单位领导及相关人员的失职责任。

各位代表，三亚的发展已经进入了一个新的历史阶段，但三亚距离“一市两都三中心”的战略目标还有相当的差距，今年改革、发展、稳定的任务更加繁重。让我们在市委的领导下，以饱满的热情、昂扬的斗志，奋发图强、扎实工作，努力实现今年的奋斗目标！
